# Bandgap engineering of tantalum nitride thin films by ion‑assisted magnetron sputtering for advanced electronic and radiation detection applications

**DOI:** 10.1007/s10854-026-18593-y

**Published:** 2026-10-07

**Authors:** Galina Nagicheva, Mikhail Vostokov, Michaël Chesaux, Jacek Wojcik, Attila Csík, Sándor Kökényesi, Dino Deligiannis, Alla Reznik

**Affiliations:** 1 Lakehead University, 955 Oliver Road, Thunder Bay, ON P7B 5E1 Canada; 2 Intlvac Thin Film Corporation, 247 Armstrong Avenue, Halton Hills, ON L7G 4X6 Canada; 3 HUN-REN Institute for Nuclear Research, Bem tér 18/c, Debrecen, 4026 Hungary; 4 Institute of Physics, University of Debrecen, Bem tér 18, Debrecen, 4026 Hungary; 5 Thunder Bay Regional Health Research Institute, 290 Munro Street, Thunder Bay, ON P7A 7T1 Canada; 6 Ontario Institute for Cancer Research, 661 University Avenue, Suite 510, Toronto, ON M5G 0A3 Canada

## Abstract

Tantalum nitride (TaN_x_) thin films exhibit a wide range of electrical and optical properties that can be controlled by adjusting deposition conditions, making them attractive for microelectronics, protective coatings, and functional layers in radiation detectors and other advanced devices. In this work, TaN_x_ films were deposited by ion‑assisted reactive magnetron sputtering while systematically varying the N_2_/Ar flow‑rate ratio. Optical transmission/reflectance spectroscopy combined with spectroscopic ellipsometry and the Tauc method analysis shows that increasing N_2_/Ar flow-rate ratio governs a continuous transition from quasi-metallic Ta-rich films to semiconducting N-rich films, with indirect optical bandgaps increasing from near zero to about 1.6 eV. This transformation is accompanied by a corresponding increase in resistivity over roughly five orders of magnitude, from ~10^–2^ to ~10^3^ Ω·cm. Raman spectroscopy and grazing-incidence X-ray diffraction reveal that all films are nanocrystalline or quasi-amorphous TaN_x_ with short-range order embedded in a disordered matrix; increasing N_2_/Ar suppressing cubic TaN-like formation and promoting nitrogen-rich, structurally disordered Ta–N configurations. Rutherford backscattering spectrometry confirms that N-rich films at N_2_/Ar ≈ 2 are compositionally uniform, with stoichiometry lying between Ta_3_N_5_ and Ta_4_N_5_. Overall, these results establish quantitative deposition–structure–property relationships in nanocrystalline TaN_x_ thin films and demonstrate that ion-assisted reactive sputtering enables effective tuning of resistivity and optical bandgap. The identified correlations are essential for optimizing electro-optical performance in low-resistivity contacts and liners, as well as high-resistivity blocking and passivation layers for electronic, photonic, and detector applications.

## Introduction

Tantalum nitride (TaN_x_) is a refractory transition-metal nitride widely used in microelectronics and related technologies, including thin-film resistors, Cu-interconnect diffusion barriers and liners, protective coatings and emerging photonic applications [1–5]. In most device‑relevant processes, TaN_x_ is obtained as a nanocrystalline or quasi‑amorphous thin film whose composition, microstructure, and resulting electro‑optical properties are primarily determined by the deposition technique and can be tuned by parameters such as nitrogen content, deposition pressure, substrate temperature, deposition power, and total gas flow [6, 7].

A variety of thin-film deposition techniques have been employed to grow TaN_x_ layers, including reactive DC (direct current) and RF (radio frequency) magnetron sputtering under combined nitrogen and argon gas flow, high-power impulse magnetron sputtering (HiPIMS), cathodic arc deposition, atomic layer deposition (ALD), and chemical vapor deposition (CVD) [4, 5, 8, 9]. Among them, reactive magnetron sputtering is particularly attractive for bandgap and resistivity control because the N_2_/Ar flow ratio, plasma conditions, and substrate temperature can be adjusted to drive TaN_x_ from Ta-rich, quasi-metallic phases to N-rich, semiconducting or insulating compositions, while maintaining compatibility with semiconductor process flows. Additionally, magnetron sputtering offers precise control over thickness, uniformity, and deposition rate, while ion assistance can improve film density and microstructure without requiring high processing temperatures.

The inherent flexibility of reactive magnetron sputtering has made it an attractive technique for the deposition of TaN_x_ thin films as diffusion-barrier and liner layers in advanced Cu-interconnect metallization for CMOS-compatible integrated circuits, as well as in power-device metallization stacks [1–3, 10, 11]. Low- to moderate-resistivity Ta-rich TaN_x_ layers are typically required to minimize series resistance and current crowding while maintaining robust suppression of Cu diffusion into surrounding dielectrics [12, 13]. In these stacks, the interplay between phase composition, nanocrystalline structure, and resistivity has a direct impact on electromigration reliability and via resistance. Beyond interconnects and power devices, the same ability to tune resistivity makes TaN_x_ a widely used material in other integrated components. In thin-film resistor and integrated-heater applications, TaN_x_ is widely used as a stable resistive material whose sheet resistance and temperature coefficient of resistance can be tailored by adjusting nitrogen content and deposition conditions, and similar TaN_x_ films are employed in electro-microfluidic and chromatographic systems as biasing electrodes and microheaters, where controllable resistivity, chemical stability, and adhesion to silicon or glass substrates are essential [14–17].

For applications that rely on controlled charge injection or leakage suppression, such as optical sensors and multilayer photoconductive structures used in direct‑conversion radiation detectors, high‑resistivity blocking or passivation layers are commonly employed to reduce dark current, improve signal‑to‑noise ratio, and protect device surfaces [18–21]. Nitrogen‑rich, nanocrystalline TaN_x_ films that combine high resistivity with structural stability and compatibility with CMOS and TFT processes can serve this purpose, provided that their bandgap, defect structure, and microstructure can be appropriately tuned.

It should be mentioned that TaN_*x*_ is not unique in its sensitivity to nitrogen incorporation. In many reactively sputtered nitride systems, changes in nitrogen incorporation substantially affect film composition, phase formation, microstructure, bonding, and electrical and optical properties. For example, in TiN_x_ films, increasing nitrogen content changes the phase composition from Ti–rich α-Ti toward Ti_2_N and, at higher nitrogen concentrations, TiN; the electrical resistivity initially increases strongly at low N content and subsequently decreases as the nitride phases become dominant [22]. Related studies of Ti–rich TiN, TiC, and TiCN films have further shown that changes in composition and local atomic coordination are accompanied by changes in bonding, structural order, defects, and phonon-related properties [23]. CrN_x_ films show a similar sensitivity to nitrogen incorporation: increasing N_2_ flow drives the composition from Cr-rich toward stoichiometric and N-rich CrN, accompanied by a strong increase in resistivity and a transition from electron-dominated to hole-dominated transport [24, 25]. Nitrogen incorporation also strongly affects phase formation and microstructure in Cr-based nitride coatings, where increasing N content promotes evolution from metallic bcc-Cr toward Cr_2_N- and CrN-containing structures together with changes in grain structure and morphology [26]. In AlN films, variation of N_2_ flow affects crystallographic orientation, film density, surface morphology, local atomic coordination, and Raman-active phonon modes, demonstrating that nitrogen incorporation can influence both long-range and short-range structural order [27].

The above examples illustrate the broad role of nitrogen incorporation in controlling the evolution of composition, local bonding, microstructure, and electrical properties in reactively sputtered nitride films. Similar trends have been observed in reactively sputtered Ta–N films, where sputtering power, substrate temperature, nitrogen partial pressure (or N_2_/Ar flow-rate ratio), and other deposition parameters have been shown to control the structure, hardness, and electrical resistivity, demonstrating that both resistivity and phase composition can be tuned over a wide range [16, 28–30]. For example, it was demonstrated that the electrical properties of sputtered TaN_x_ films can be tuned over several orders of magnitude by varying nitrogen content and deposition conditions. Hu et al. reported that resistivity varied by approximately three orders of magnitude with increasing N_2_ flow rate, accompanied by corresponding changes in optical constants [31]. Li et al. observed an almost two-order-of-magnitude increase in sheet resistance with nitrogen flow ratio, further confirming the strong sensitivity of TaN_x_ electrical properties to nitrogen incorporation [16]. Recent work by Su et al. also showed composition-dependent resistivity variations of nearly three orders of magnitude and linked these changes to secondary electron emission behavior [29]. Studies have also reported transitions between amorphous, nanocrystalline, and columnar microstructures as a function of deposition parameters [7, 32–35]. Optical studies of Ta (oxy) nitrides have shown that nitrogen-rich compositions can exhibit semiconducting behavior with effective bandgaps in the visible range [32, 36–38].

Overall, the demonstrated tunability in structural and electro-optical properties of TaN_x_ films is an attractive advantage of this material. However, full utilization of this advantage requires establishing the simultaneous correlation of optical bandgap, electrical resistivity, local bonding disorder, and composition as a function of deposition conditions, which we address in the present work.

Specifically, we investigate TaN_x_ thin films deposited by ion-assisted reactive magnetron sputtering and show how the N_2_/Ar flow-rate ratio affects the structural, optical, and electrical properties of the films. We demonstrate continuous control of the optical bandgap from near-zero in Ta-rich, quasi-metallic films to approximately 1.6 eV in nitrogen-rich, semiconducting films, accompanied by a corresponding change in resistivity spanning five orders of magnitude (from ~10^–2^ to ~10^3^ Ω·cm). Investigation of corresponding changes in structural and morphological properties suggests that this transition is governed predominantly by changes in short-range order, local Ta-N bonding, and disorder in nanocrystalline/quasi-amorphous films, rather than by well-defined phase transitions. The demonstrated results establish quantitative deposition-structure–property relationships and enable technology optimization for controlled fabrication of low-resistivity contacts and liners or high-resistivity blocking and passivation layers for electronic and radiation-detector applications.

## Materials and methods

### TaN_x_ thin film deposition

Tantalum nitride (TaN_x_) thin films were deposited using a Nanochrome IV PARMS (Plasma-Assisted Reactive Magnetron Sputtering) system from Intlvac operated in dual-cathode AC. The deposition chamber was evacuated to a base pressure of 1.5 × 10^–6^ Torr. A tantalum target (diameter = 15 cm, thickness = 0.6 cm, purity = 99.9%) served as the sputtering source.

Depositions were performed on two types of substrates:

(100) Silicon wafers (diameter = 100 mm, thickness = 525 μm) for electrical, structural, and hardness measurements.
UV-grade fused silica substrates (diameter = 25 mm, thickness = 1.5 mm) for optical measurements (transmission, reflectance, and bandgap determination).

All depositions were performed using the 20 cm-diameter rotating substrate holder. A fused-silica witness sample was placed at the center of the holder, while silicon wafers were positioned symmetrically around it at equal distances. The deposition rates varied from approximately 0.5 to 1.2 Å/s for different N_2_/Ar gas flow ratios resulting in TaN_x_ layers’ thicknesses, *d,* in the range of 190–430 nm.

During deposition, the sputtering power and substrate temperature were maintained at 1000 W and 150 °C, respectively, while the N_2_/Ar flow-rate ratio was systematically varied from 0.5 to 3.0.

Thickness and uniformity of TaN_x_ were determined using a KLA P-16 surface profilometer. Measurements were performed across the wafer diameter at ~1 cm intervals, with a stylus force of 2 mg and total scan distance of 600 μm.

### Morphological and structural characterization

Surface morphology of the deposited TaN_x_ films was examined using scanning electron microscopy (SEM). The measurements were performed with a Thermo Scientific Scios field-emission scanning electron microscope operated at an accelerating voltage of 2 kV and a beam current of 0.20 nA using the T2 detector. Images were acquired at a magnification of 35,000×, corresponding to a horizontal field width of approximately 3.6 μm. The SEM analysis was used to evaluate the surface morphology, film continuity, and qualitative grain structure of the TaN_x_ films.

Raman spectroscopy was employed to probe local bonding configurations and short‑range structural order in the TaN_x_ films. Raman measurements were conducted using an in Via Raman microscope (Renishaw, UK) equipped with a 2400 lines mm^⁻1^ diffraction grating and providing a spectral resolution of approximately 1 cm^⁻1^. Prior to data collection, the spectrometer calibration was verified using the characteristic silicon Raman band at 520 cm^⁻1^. The spectra were obtained from the surface of the TaN_x_ films under 532 nm laser excitation. The laser power was carefully minimized during acquisition to prevent thermal effects or photo-induced structural modifications, such as crystallization or phase transitions within the films [39, 40].

The long‑range structural properties and phase composition of the TaN_x_ films were investigated by grazing-incidence X-ray diffraction (GIXRD). Diffraction measurements were carried out at a fixed incidence angle (ω = 0.7°), while the diffracted intensity was collected over a 2θ range of 20–80° at room temperature. Grazing-incidence geometry was used to enhance surface sensitivity and suppress contributions from the silicon substrate.

Rutherford backscattering spectrometry (RBS) was used to determine the elemental composition and thickness of the TaN_x_ films deposited on Si substrates. Measurements were performed using a 3.0 MeV He^+^ ion beam at normal incidence (0°) with a scattering angle of 170°, and a total accumulated charge of approximately 6.0 μC. The backscattered ions were detected with a solid-state detector positioned at the scattering angle, and the spectra were recorded as ion yield versus channel number (energy). Experimental spectra were analyzed using the MEISwin simulation software, which was used to reproduce the measured spectra and extract the elemental depth profiles of Ta, N, O, Ar, and Si. The simulations were performed assuming a TaN layer density of 11.44 g cm^⁻3^ (≈80% of the bulk TaN density of 14.3 g cm^⁻3^, as described in [41]). In the simulations, film thickness was obtained from the calculated areal atomic density (atoms cm^⁻2^) and converted to physical thickness using the assumed material density.

### Optical characterization and bandgap determination

Optical transmittance (T) and reflectance (R) of the TaN_x_ films deposited on UV-grade fused-silica substrates were measured using a Cary UV–vis-NIR spectrophotometer. Prior to each measurement, baseline correction was performed to eliminate instrumental background contributions. Spectra were recorded over the wavelength range of 350–2500 nm with a scan rate of 150 nm min^⁻1^. The optical losses (absorption) were evaluated using the relation

1 $$A \, = \, 1 \, {-} \, T \, {-} \, R$$

From the measured T and R spectra, the absorption coefficient (α) was calculated using the single-slab model that accounts for multiple reflections at both interfaces [42, 43]. When *R*_*meas*_^*2*^*exp(− 2αd)* << 1, α was obtained from.

2 $$\alpha =-\frac{1}{d}ln\left(\frac{{T}_{meas}}{{\left(1-{R}_{meas}\right)}^{2}}\right)$$

where *d* is the film thickness.

The calculated absorption coefficient was then used to determine the optical bandgap (*E*_*g*_) according to the Tauc method [44–46], which assumes that the absorption coefficient near the band edge follows

3 $$\alpha h\nu =const.{\left(h\nu -{E}_{g}\right)}^{n}$$

where *hν* is the photon energy, and *n* is the transition exponent (*n* = *2* for indirect and *n* = *0.5* for direct allowed transitions).

Tauc plots of (αhν)^1/n^ vs. *hν* were constructed, and ${E}_{g}$ was obtained by linear extrapolation of the linear region to the x-axis intercept.

Likewise, variable-angle spectroscopic ellipsometry (VASE) measurements were performed to determine the optical constants and optical bandgap of the TaN_x_ thin films. The ellipsometry parameters Ψ and Δ were measured over the spectral range of 245–1688 nm at five angles of incidence (55°, 60°, 65°, 70°, and 75°) using an M-2000 spectroscopic ellipsometer. The data were analyzed using CompleteEASE software (J.A. Woollam Co., Inc.) by fitting a multilayer optical model consisting of a silicon substrate, a native SiO_2_ layer, and the TaN_x_ film, with surface roughness represented using an effective medium approximation (EMA).

For TaN_x_ films deposited at N_2_/Ar ≥ 1.5, the complex dielectric function of the TaN_x_ layer was parameterized using Cody–Lorentz oscillator within the General Oscillator (Gen-Osc) model [47–49]. The film thickness, surface-roughness thickness, and optical-model parameters were varied during analysis to obtain the best simultaneous fit to the experimental Ψ and Δ spectra measured at all five angles of incidence. The refractive index (*n*) and extinction coefficient (*k*) were subsequently obtained from the fitted dielectric function, and the optical bandgap (*E*_*g*_) was determined from the Cody–Lorentz model. For films deposited at N_2_/Ar < 1.5, the optical response was strongly metallic, with substantial absorption extending throughout the spectral range investigated; consequently, a reliable optical bandgap could not be determined from the ellipsometry data. Good agreement between the experimental and modeled Ψ and Δ spectra was obtained for all analyzed samples, with mean squared error (MSE) values below 5.

### Resistivity measurements

The sheet resistance of the TaN_x_ films was measured using both four-point and two-point probe techniques. For the four-point measurements, a Cascade C45 54/S probe head (osmium tips, 1 mm spacing) was mounted on a microscope translation stage. The outer probes were connected to the output terminals of a BK Precision 1787B DC power supply, while the current was measured using a Brymen BM257s multimeter. The voltage drop between the inner probes was measured using an HP 34401A multimeter. The applied voltage during measurements was 5 V. The setup provided simultaneous readings of current (I) and voltage (V) at the film surface. The sheet resistance *R*_*s*_ was calculated using the standard relation ${R}_{s}=\left(\frac{\pi }{\mathrm{l}\mathrm{n}(2)}\right)\left(\frac{V}{I}\right)$ was used, and the electrical resistivity was obtained from $\rho ={R}_{s}\cdot d$, where *d* is the film thickness [50, 51]. Because of the measurable range of this method, the four-point probe was primarily used for low nitrogen-content films (N_2_/Ar = 0.5 and 1), which exhibited relatively low resistivity. For nitrogen-rich TaN films, where resistances were significantly higher, a DESCO two-point surface resistance measurement system was employed, operating over a range of 10^3^–10^12^ Ω. This digital resistance meter directly provided the surface resistance of the film, which was then converted to resistivity using the same relation as above.

## Results

### Morphological and structural properties

**Fig. 1:**
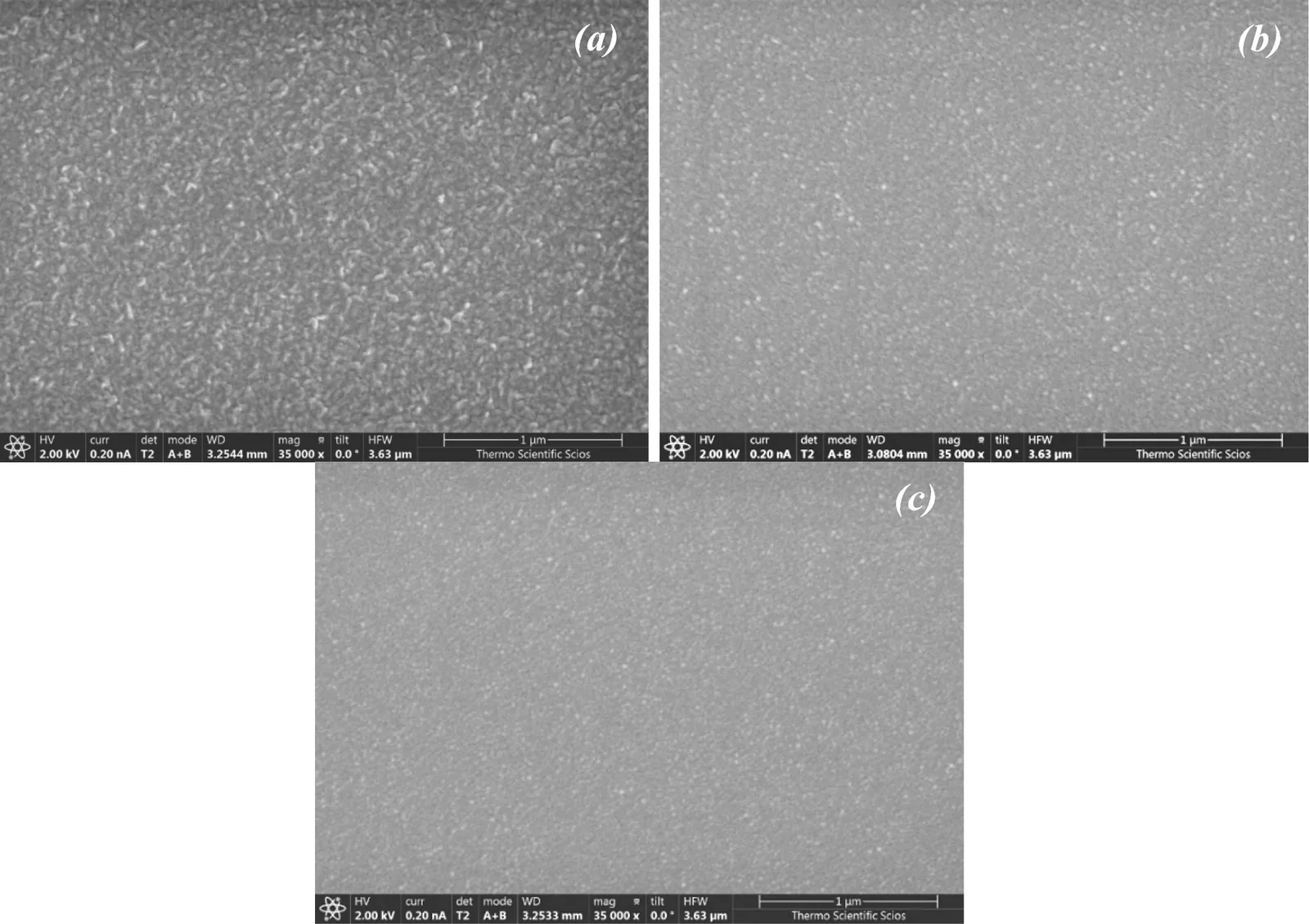
SEM images showing the surface morphology of TaN films deposited at **a** N_2_/Ar = 0.5 (*d* = 430 nm), **b** 1.5 (*d* = 235 nm), and **c** 3.0 (*d* = 190 nm)

The surface morphology of the TaN_x_ films deposited at different N_2_/Ar ratios is shown in Fig. 1. All films exhibit homogeneous and uniform surfaces without visible cracks and voids. No pronounced faceted grains are observed, indicating a fine-grained or nanocrystalline microstructure. The film deposited at N_2_/Ar = 0.5 (*d* = 430 nm) shows relatively higher surface contrast and more pronounced morphological variations, suggesting greater surface heterogeneity. As the N_2_/Ar ratio increases to 1.5 (*d* = 235 nm) and 3 (*d* = 190 nm), the surface becomes progressively smoother and more homogeneous, with finer apparent surface features. Because individual nanocrystalline grains cannot be reliably resolved in the present SEM images, these observations are described as morphological refinement rather than as a quantitative reduction in grain size. Overall, the SEM observations indicate that higher nitrogen fractions lead to finer surface morphology while maintaining dense and continuous film coverage.

**Fig. 2:**
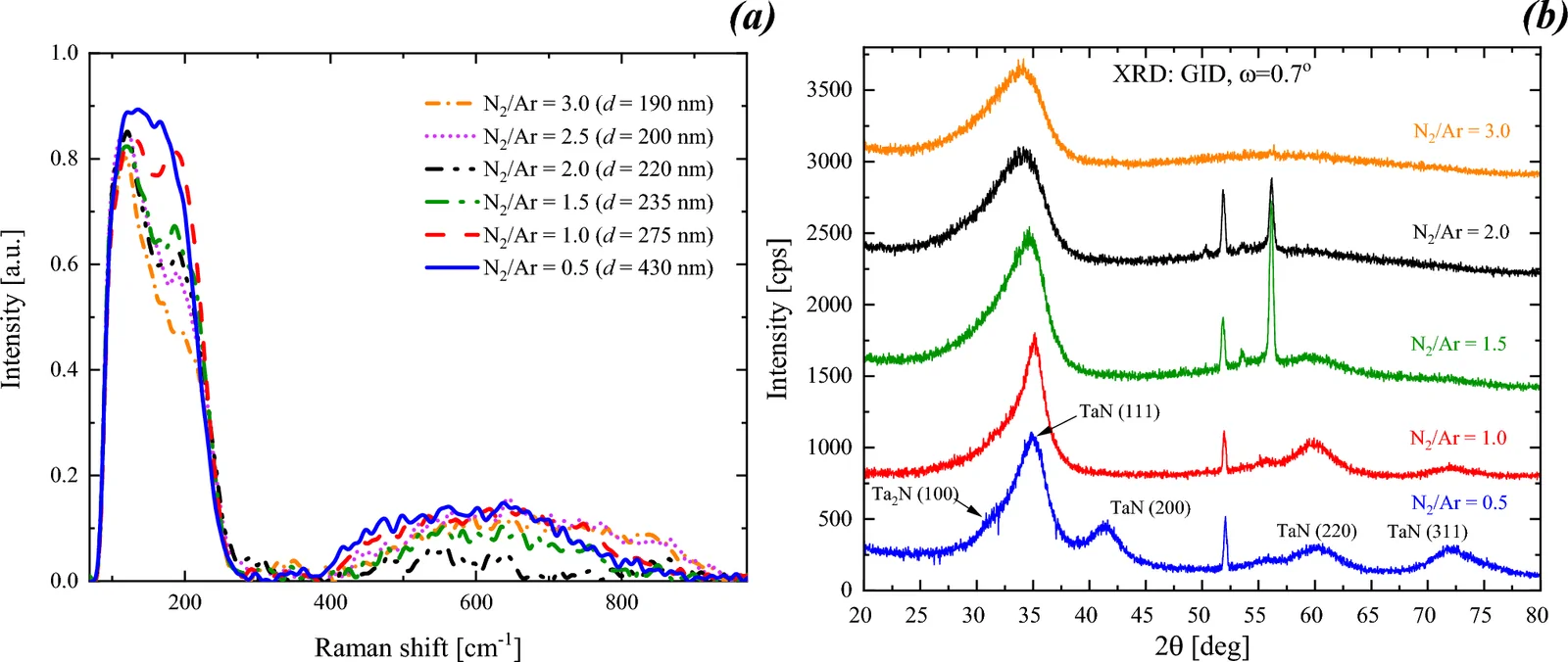
**a** Raman spectra and **b** grazing-incidence diffraction (GID) patterns of TaN_x_ films deposited at different N_2_/Ar flow ratios

The Raman spectra of the TaN_x_ films (Fig. 2a) deposited under different sputtering conditions a structurally disordered Ta–N system with predominantly short-range order. All samples exhibit a broad low-frequency band in the 100–250 cm^−1^ range. At the lowest N_2_/Ar ratio of 0.5, the Raman response in this range appears as a single broad peak centered below 200 cm^−1^, consistent with overlapping acoustic-like Ta–N vibrational contributions reported for transition-metal nitride systems [52]. With increasing N_2_/Ar ratio, this band gradually evolves into two partially resolved peaks near 120 and 200 cm^−1^; the relative intensity of the ~120 cm^−1^ peak increases with increasing N_2_/Ar ratio, consistent with an increased occupation of interstitial nitrogen sites, as previously reported [53–55], while that of the ~200 cm^−1^ peak decreases, and at N_2_/Ar ≈ 3 the higher-frequency ~200 cm^−1^ peak is no longer distinguishable from the underlying broad band. The redistribution of intensity between these components is interpreted as evidence of changes in the local Ta–N coordination environment and short-range structural order with increasing nitrogen incorporation, within a disordered TaN_x_ matrix.

In addition, a very broad spectral contribution extending from approximately 400 to 800 cm^⁻1^ is observed in most samples. This high-frequency continuum is characteristic of defect-assisted and/or second-order Raman scattering in disordered transition-metal nitrides [53, 54]. Although this spectral region overlaps with Raman bands reported for tantalum oxides, the absence of distinct, well-resolved oxide-related peaks indicates that the observed signal does not arise from a well-defined crystalline oxide phase. Overall, the broadening and redistribution of Raman intensity with increasing N_2_/Ar ratio are interpreted as evidence of changes in local Ta–N bonding and increasing structural disorder rather than of phase transformations. The grazing-incidence X-ray diffraction (GID) data (Fig. 2b) exhibit a broad diffraction peak centered at 2θ ≈ 34–35° for all N_2_/Ar gas flow ratios. With increasing nitrogen content, this feature becomes progressively broader and its intensity decreases, indicating increasing structural disorder and reduced long-range crystallinity. The position of this broad feature overlaps with reflections reported for several Ta–N phases, including diffraction peaks reported for the (111) planes of both cubic B1-type TaN and hexagonal Ta_5_N_6_ [16, 32, 52, 54–56]. Thus, this feature cannot be uniquely assigned to a single crystalline phase.

For the film deposited at N_2_/Ar = 0.5, additional weak diffraction peaks are observed at approximately 42°, 60°, and 72°. These peak positions are consistent with the (200), (220), and (311) reflections commonly reported for cubic TaN-like ordering [16, 33, 53, 55–57], although contributions from other Ta–N phases cannot be excluded because of the broad and overlapping nature of the diffraction features. The intensity of these peaks decrease with increasing N_2_/Ar ratio and they are no longer detectable at N_2_/Ar = 2. In addition, sharp peaks are observed near 52° and 56°. The peak near 52° is consistent with hexagonal TaN (201) [58], while the peak near 56°, detected only for N_2_/Ar = 1.5 and 2, may be associated with tetragonal TaN_1.86_ (112) [59]. These narrow peaks indicate the presence of minor crystalline Ta–N domains included in the predominantly disordered/nanocrystalline matrix, rather than a fully developed crystalline phase. At N_2_/Ar = 3, these sharp features disappear, suggesting that further nitrogen incorporation suppresses long-range crystalline ordering and promotes a more disordered nitrogen-rich Ta–N structure. Overall, the GIXRD data therefore support a predominantly disordered/nanocrystalline TaN_x_ matrix with minor crystalline contributions whose relative prominence depends on the N_2_/Ar ratio. 

**Fig. 3:**
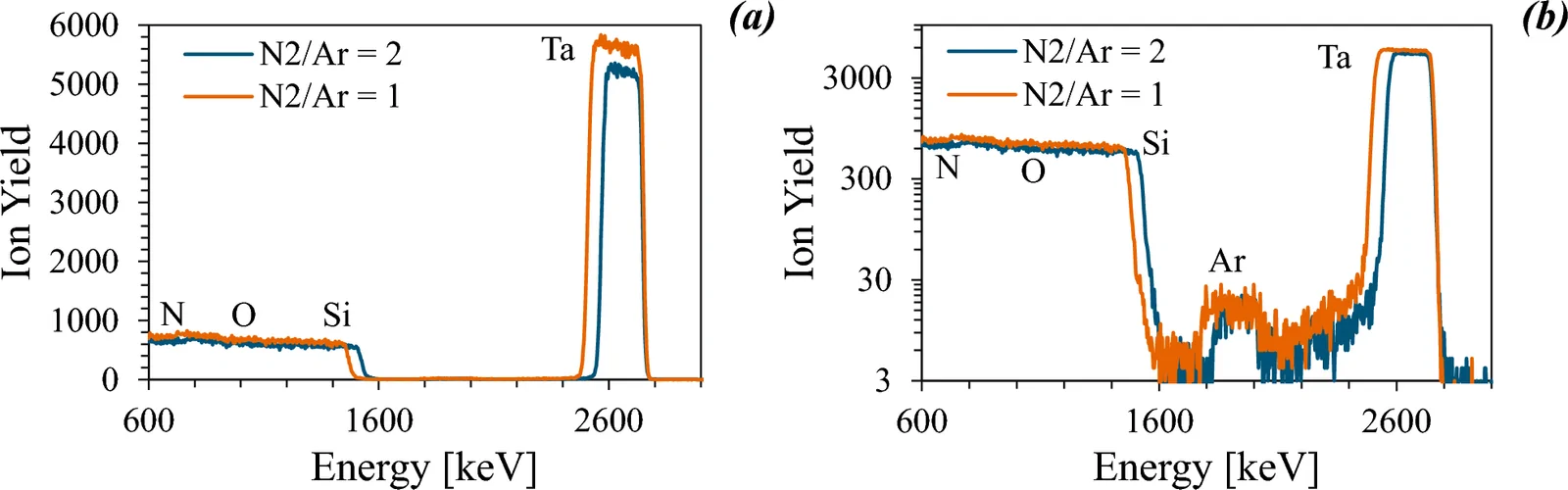
RBS spectra of TaN_x_ shown in linear **a** and logarithmic **b** ion-yield scales. For N_2_/Ar = 2, the composition used in the simulation was Ta_0.38_N_0.54_O_0.05_Ar_0.018_, which lies approximately between the stoichiometries Ta_3_N_5_ and Ta_4_N_5_. For N_2_/Ar = 1, the composition used for the simulation (Ta_0.42_N_0.50_O_0.05_Ar_0.019_) is closer to Ta_4_N_5_

**Fig. 4:**
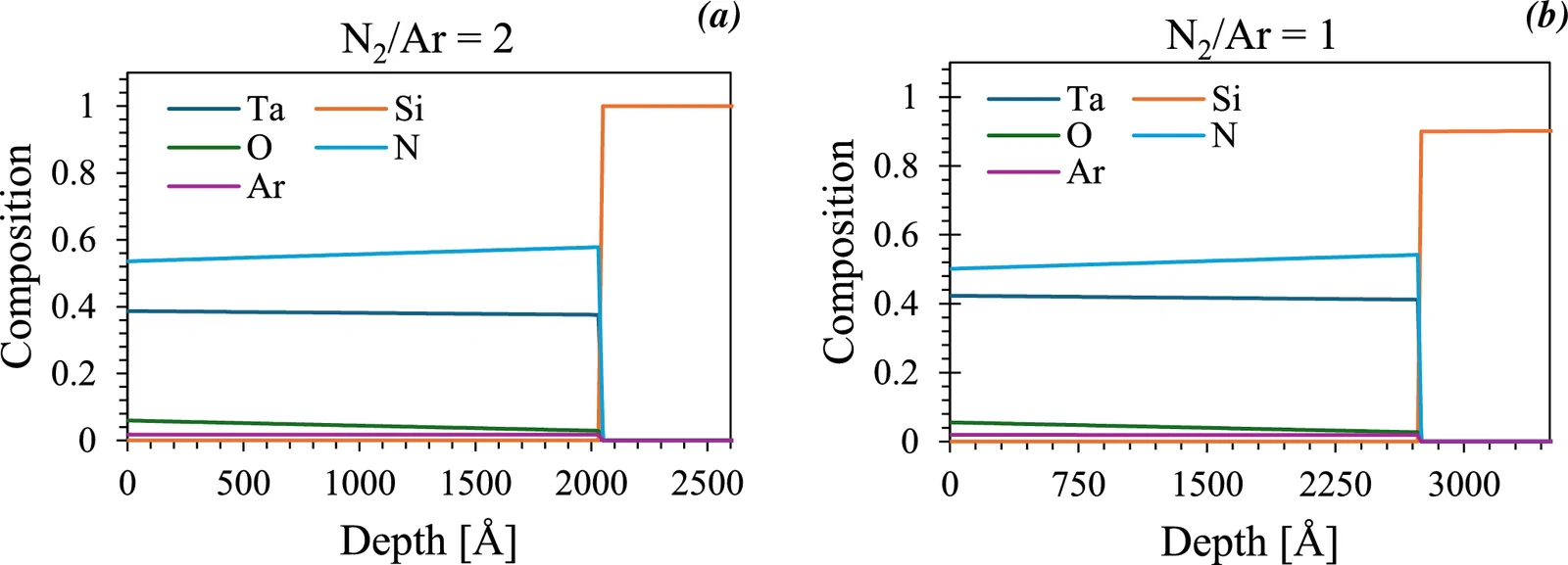
The RBS-simulated depth profiles for N_2_/Ar = 2 **a** and 1 **b**. Thicknesses were calculated using a density of 11.44 g/cm^3^ for the TaN layer (the bulk density is 14.3 g/cm^3^, and 11.44 g/cm^3^ corresponds to 80% of the bulk density)

Rutherford backscattering spectroscopy confirms the formation of dense TaN_x_ films with compositions that depend on the N_2_/Ar ratio during deposition (Figs. 3 and 4). For N_2_/Ar = 1, the best-fit RBS simulation yielded an approximate composition of Ta_0.42_N_0.50_O_0.05_Ar_0.019_, which is close to the Ta_4_N_5_ configuration. Increasing the N_2_/Ar ratio to 2 results in a lower Ta fraction and a higher N/Ta ratio, with an approximate best-fit composition of Ta_0.38_N_0.54_O_0.05_Ar_0.018_. This composition lies between the Ta_4_N_5_ and Ta_3_N_5_ stoichiometries, suggesting a trend toward a more nitrogen-rich, or Ta-deficient, Ta–N phase at higher nitrogen flow. These compositions should be regarded as approximate best-fit stoichiometries rather than exact atomic ratios, since the determination of light elements, particularly nitrogen and oxygen, carries relatively large uncertainty in RBS due to their weak scattering signals and partial overlap with the Si substrate contribution. Within these limits, the RBS analysis nonetheless supports a systematic trend toward nitrogen enrichment with increasing N_2_/Ar ratio.

The corresponding depth profiles show nearly uniform elemental distributions across the film thickness, with a slight increase in nitrogen content toward the film–substrate interface for both compositions. This indicates mostly homogeneous growth without significant compositional gradients (Fig. 4). The film thickness obtained from RBS simulations was approximately 278 nm for N_2_/Ar = 1 and 205 nm for N_2_/Ar = 2, which is in good agreement with the thickness measured by profilometry (275 nm and 220 nm, respectively).

### Electro-optical properties

**Fig. 5:**
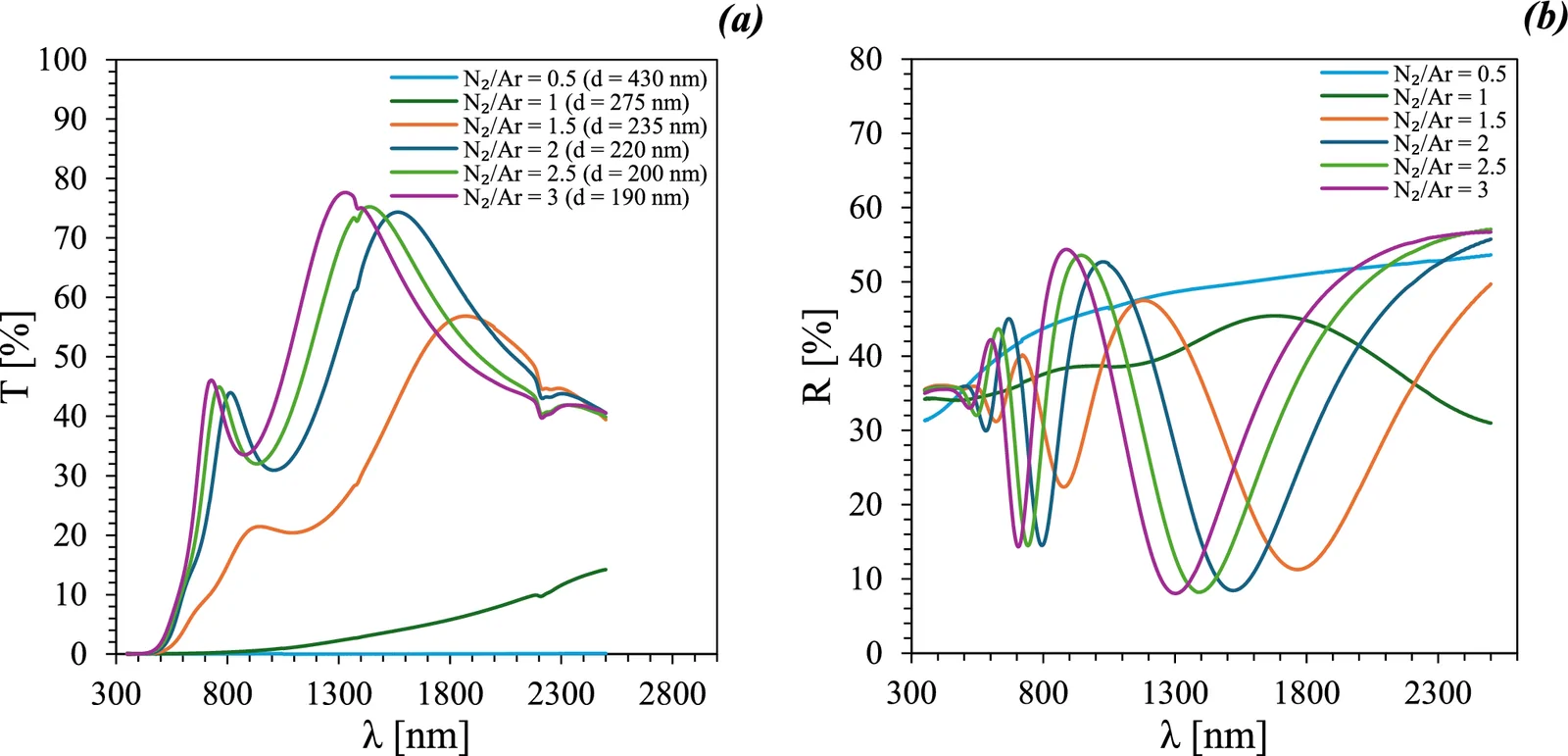
Optical transmission **a** and reflectance **b** spectra of TaN_x_ films deposited at varying N_2_/Ar flow ratios

Transmission and reflectance spectra (Fig. 5) show that higher nitrogen content leads to a gradual increase in transmission over the visible and near-infrared range, consistent with suppression of metallic absorption and the emergence of semiconducting optical response. For N_2_/Ar ≥ 2, pronounced interference fringes appear in both T and R, indicating multiple internal reflections within the film.

The optical absorption coefficient was calculated from the measured transmittance and reflectance using Eq. (2). The applicability of this expression was numerically verified within the spectral regions selected for Tauc analysis by confirming that *R*_*meas*_^*2*^*exp(− 2αd)* << 1. For the nitrogen-rich semiconducting films *R*_*meas*_^*2*^*exp(− 2αd)* remained below approximately 5 × 10^–3^ in the selected fitting regions, indicating that the contribution of higher-order internal reflections was negligible for the optical bandgap determination. The calculation of the absorption coefficient assumes that attenuation arises predominantly from absorption. Because diffuse scattering was not measured independently, surface roughness and morphology may introduce some uncertainty into the absolute absorption coefficient.

Both direct- and indirect-bandgap Tauc analyses were performed for the nitrogen-rich semiconducting TaN_*x*_ films. The Tauc plots corresponding to the indirect transition provided the most clearly defined linear fitting region (R^2^ ≥ 99%) and therefore were used to estimate the optical gap. [60] For the films deposited at an N_2_/Ar ratios of 0.5 and 1, it was not possible to reliably determine the bandgap, as the optical response exhibited a more metallic character without a well-defined absorption edge. At higher N_2_/Ar ratios, well-defined indirect bandgaps emerge and increase from about 1.52 eV at N_2_/Ar ≈ 1.5 to about 1.64 eV at N_2_/Ar = 3 as obtained by linear extrapolation of the $\left(\alpha h\nu {)}^{1/2}\right.$ curves. Considering the disordered nanocrystalline structure of the present TaN_x_ films, these values should be regarded as estimates of the optical-gap values rather than transport-relevant mobility gap values.

**Fig. 6:**
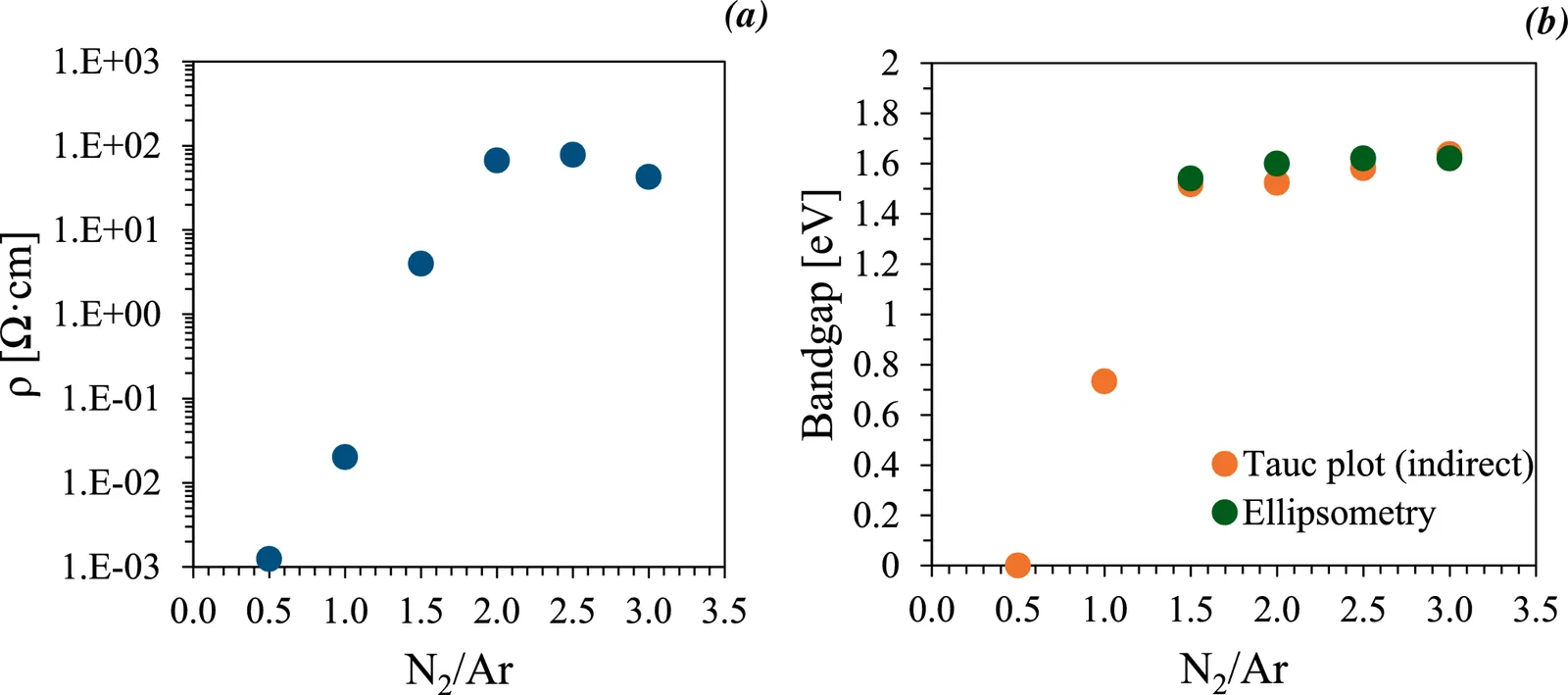
**a** Electrical resistivity (ρ) of TaN_x_ films as a function of the N_2_/Ar flow-rate ratio, **b** Optical bandgap as a function of N_2_/Ar ratio obtained from Tauc analysis and spectroscopic ellipsometry

The optical gaps derived from the Tauc plots are in reasonable agreement with those obtained independently from spectroscopic ellipsometry using a Cody–Lorentz oscillator model (Fig. 6b). This agreement supports the reliability of the extracted optical-gap values and the observed evolution of the optical gap with increasing N_2_/Ar ratio. The slight further increase in bandgap at higher N_2_/Ar ratios for nitrogen-rich films may indicate a narrowing of the band-tail region associated with shallow localized states near the band edges.

The dependence of electrical resistivity on the N_2_/Ar ratio (Fig. 6a) broadly follows the evolution of the optical gap. At low nitrogen flow (N_2_/Ar < 1), the films are Ta-rich and metallic, showing very low resistivity (~ 10^⁻2^ Ω cm). Increasing nitrogen fraction to 1–1.5 leads to formation of TaN with higher resistivity (~ 10^2^ Ω cm). Further increase (N_2_/Ar > 2) produces over-stoichiometric, N-rich phases (Ta_3_N_5_-like), which are more semiconducting, but excessive nitrogen can introduce defects, thus slightly lowering resistivity again.

## Discussion

The combined structural, morphological, and electro‑optical responses of the TaN_x_ films show a strong dependence on the N_2_/Ar ratio, reflecting systematic changes in stoichiometry, and microstructure (Table 1). At low N_2_/Ar (< 1), the films are relatively Ta-rich, with compositions approaching metallic Ta-N -like configurations, characterized by high free-carrier density and low resistivity (~ 10^–2^ Ω·cm). With increasing N_2_/Ar, the composition progressively shifts toward nitrogen-rich TaN_x_, as confirmed by Rutherford backscattering spectroscopy. For N_2_/Ar = 1, RBS simulations yield a composition close to the Ta_4_N_5_ configuration. Increasing the N_2_/Ar ratio to 2 results in a more nitrogen-rich composition which lies between the Ta_4_N_5_ and Ta_3_N_5_ stoichiometries. These stoichiometries should be regarded as approximate best-fit values rather than exact atomic ratios because of the limited sensitivity of RBS to light elements such as nitrogen and oxygen; within these uncertainties, the data nonetheless support a monotonic nitrogen enrichment with increasing N_2_/Ar. Although these compositions correspond to nitrogen-rich, or Ta-deficient, Ta-N phase, the significant peak broadening observed in GIXRD indicates that the films do not form well-defined single crystalline phases but instead remain structurally disordered nanocrystalline TaN_x_.

**Table 1:** Summary of composition, structural characteristics, electrical resistivity, and optical bandgap of TaN_x_ films deposited at different N_2_/Ar flow-rate ratios

N_2_/Ar	Deposition rate (Å/s)	d (nm)	RBS composition	Ta/N	ρ (Ω·cm)	E_g_^a^ (eV)	Structural characteristics
0.5	1.19	430	–	–	1.2 × 10^–3^	–	TaN-like nanocrystalline structure; weak cubic TaN reflections
1.0	0.76	275	Ta_0.42_N_0.50_O_0.05_Ar_0.019_	0.84	2.0 × 10^–2^	0.73	Disordered nanocrystalline TaN_x_; composition close to Ta_4_N_5_
1.5	0.65	235	–	–	4.0	1.52	Disordered TaN_x_; minor crystalline Ta–N features
2.0	0.61	220	Ta_0.38_N_0.54_O_0.05_Ar_0.018_	0.70	6.7 × 10^1^	1.52	N-rich disordered TaN_x_; composition between Ta_4_N_5_ and Ta_3_N_5_
2.5	0.56	200	–	–	7.8 × 10^1^	1.58	Predominantly disordered nanocrystalline TaN_x_
3.0	0.52	190	–	–	4.3 × 10^1^	1.64	Highly disordered N-rich TaNx; sharp crystalline features absent

^a^Optical bandgap obtained from Tauc analysis

The evolution of the indirect optical bandgap from near-zero at low N_2_/Ar to ~1.6 eV at N_2_/Ar ≥ 1.5 follows this compositional trend toward N-rich TaN_x_. Increasing nitrogen incorporation leads to the emergence of semiconducting behavior and widening the optical gap. The very slight further increase in apparent bandgap at the highest N_2_/Ar values can be associated with some reduction in band-tail absorption associated with shallow localized states near the band edges, rather than a dramatic change in the fundamental gap. 

Raman spectroscopy and grazing-incidence X-ray diffraction together provide a consistent picture of the structural evolution with N_2_/Ar ratio. Raman spectra at all N_2_/Ar exhibit broad, disorder-activated Ta-N vibrational bands and a high-frequency continuum characteristic of structurally disordered transition-metal nitrides. In GIXRD, the dominance of a single broad low-angle peak combined with the disappearance of higher-order cubic TaN diffraction peaks at elevated N_2_/Ar ratios indicates that long-range cubic TaN-like coherence is progressively lost as the films become more nitrogen-rich. This combination of broad Raman bands and broadened TaN-like diffraction peaks is characteristic of disordered nanocrystalline TaN_x_ with predominantly short-range order, rather than distinct, well-separated crystalline TaN or Ta_5_N_6_ phases. These structural trends are further supported by the SEM results. All films exhibit dense and continuous surfaces; however, the apparent grain size decreases with increasing N_2_/Ar ratio, indicating that nitrogen incorporation suppresses grain growth during deposition. The resulting microstructural refinement increases the total grain-boundary area within the films. Grain boundaries introduce structural defects and localized states that can act as both scattering centers and trapping sites for charge carriers, reducing their effective mobility and contributing to the observed increase in electrical resistivity with increasing nitrogen content [61, 62]. Consequently, the progressive transition from quasi-metallic to semiconducting transport behavior can be attributed to the combined effects of reduced carrier concentration associated with nitrogen-rich bonding and enhanced carrier scattering at grain boundaries in these disordered nanocrystalline TaN_x_ films.

From an application perspective, the property map established here demonstrates that ion-assisted reactive magnetron sputtering enables the fabrication of TaN_x_ films spanning a wide range of electrical and optical properties within a single deposition platform. Ta-rich films deposited at low N_2_/Ar ratios exhibit low resistivity suitable for conductive layers and contacts, whereas nitrogen-rich TaN_x_ films deposited at higher N_2_/Ar ratios exhibit higher resistivity and wider bandgaps, making them attractive for blocking or passivation layers in electronic and radiation-detector devices. The ability to tune these properties through controlled adjustment of the N_2_/Ar ratio highlights the versatility of nanocrystalline TaN_x_ as a functional material system for electronic and photonic applications. 

## Conclusions

The combined structural, compositional, optical, and electrical characterization demonstrates that the properties of ion-assisted reactively sputtered TaN_x_ films can be systematically tuned by varying the N_2_/Ar flow ratio. Across the investigated deposition conditions, the films exhibit a disordered nanocrystalline structure. Increasing the N_2_/Ar ratio drives a transition from Ta-rich, low-resistivity films with quasi-metallic behavior to N-rich, high-resistivity semiconducting films with well-defined indirect optical bandgaps of up to ~1.6 eV. These results establish deposition–structure–property relationships for TaN_x_ and define a practical process space in which Ta-rich films are suited for conductive contacts and liners, whereas N-rich TaNx films are better suited for high-resistivity blocking or passivation layers in electronic and radiation-detector devices.

## Data Availability

Data sets generated during the current study are available from the corresponding author on reasonable request.
